# Pyroglutamate Aβ cascade as drug target in Alzheimer’s disease

**DOI:** 10.1038/s41380-021-01409-2

**Published:** 2021-12-08

**Authors:** Thomas A. Bayer

**Affiliations:** Department of Psychiatry and Psychotherapy, Division of Molecular Psychiatry, University Medical Center Göttingen (UMG), Georg-August-University Göttingen, 37075 Göttingen, Germany

**Keywords:** Drug discovery, Neuroscience

## Abstract

One of the central aims in Alzheimer’s disease (AD) research is the identification of clinically relevant drug targets. A plethora of potential molecular targets work very well in preclinical model systems both in vitro and in vivo in AD mouse models. However, the lack of translation into clinical settings in the AD field is a challenging endeavor. Although it is long known that N-terminally truncated and pyroglutamate-modified Abeta (Aβ_pE3_) peptides are abundantly present in the brain of AD patients, form stable and soluble low-molecular weight oligomers, and induce neurodegeneration in AD mouse models, their potential as drug target has not been generally accepted in the past. This situation has dramatically changed with the report that passive immunization with donanemab, an Aβ_pE3_-specific antibody, cleared aymloid plaques and stabilized cognitive deficits in a group of patients with mild AD in a phase II trial. This review summarizes the current knowledge on the molecular mechanisms of generation of Aβ_pE_, its biochemical properties, and the intervention points as a drug target in AD.

## Introduction

For more than three decades, the amyloid cascade hypothesis [1] claiming that amyloid (Aβ) peptides as cleavage products of the precursor amyloid precursor protein (APP) [2] and accumulation in plaques triggers neuron loss and neurofibrillary tangle formation. Since the first report on the peptide sequence of Aβ_1-24_ of the N-terminus isolated from cerebrovascular amyloid preparations from Alzheimer’s disease (AD) and Down syndrome brain [3] followed by peptide sequencing of amyloid-β isolated from plaques it became evident that amyloid in plaque cores must be predominantly N-truncated [4]. Later, Mori et al. [5] described an N-truncated Aβ variant starting with pyroglutamate at position 3 (Aβ_pE3-*X*_). Saido et al. [6] was the first to compare the staining pattern of Aβ_1-*X*_ with Aβ_pE3-*X*_ specific antibodies and showed that the N-truncated Aβ variant is a dominant fraction in plaques in AD brain. Russo et al. [7] reported that both Aβ_1-*X*_ and Aβ_pE-*X*_ can form stable water-soluble aggregates. Using matrix-assisted laser-desorption-time-of-flight mass spectrometry a variety of N-truncated Aβ peptides, including Aβ_pE3-*X*_ in amyloid plaques very identified [8]. Wildburger et al. [9] used high-resolution mass spectrometry and identified a wide range of N- and C-truncated amyloid-β peptides from post-mortem brain of AD patients demonstrating no correlation with post-mortem interval. Portelius et al. [10] revealed the relative abundance of full-length and N-truncated variants using immunoprecipitation in combination with mass spectrometric analysis in different brain areas of sporadic and familial AD cases. The major variants were Aβ_1-42_, Aβ_pE3-42_, Aβ_4-42_ followed by Aβ_1-40_ in both sporadic and familial AD cases. Moreover, Upadhaya et al. [11] demonstrated that Aβ_pE3-*X*_ western blotting can be used as an informative biomarker for biochemical amyloid-β staging in post-mortem brain tissue from symptomatic AD and preclinical AD cases. Besides Aβ_pE-*X*_ and a phosphorylated Aβ variant found in plaques, they were also observed as soluble aggregates in a disease-specific manner. The authors concluded that the level of different Aβ variants occur in a hierarchical sequence allowing the distinction of three biochemical amyloid-β stages, with stage 1 and its characteristic marker Aβ_1-*X*_, followed by stage 2 with Aβ_pE3-*x*_ and stage 3 with a phosphorylated Aβ [11]. In good agreement, Moro et al. [12] observed that deposition of Aβ_pE-*X*_ is closely related to AD, but not with normal ageing and is found in plaques and neurons. In AD mouse models, similar observations were observed. In APP23 mouse brain, Aβ_pE-*X*_ deposits appear during ageing as a maturation process of amyloid plaques starting with Aβ_1-*X*_ first followed by Aβ_pE3-*X*_ deposits [13]. In APP/PS1KI mice, Aβ_pE3-*X*_ positive plaques increased with age, on the expense of Aβ_1-*X*_ [14]. Transgenic mice expressing mutant Aβ_Q3-42_ elicit partial conversion of N-terminal Gln-3 into pyroglutamate Glu-3 in a cell-type dependent manner depending on the different mouse line likely due to specific genomic integration of the transgene. Besides abundant loss of Purkinje cells and ataxia [15], degeneration of hippocampus CA1 neurons and cognitive decline [16], or loss of striatal neurons associated with basal locomotor activity and sensorimotor gating (when coexpressed with human QC) [17] was reported in different transgenic lines.

## Generation of pyroglutamate Aβ

**Fig. 1:**
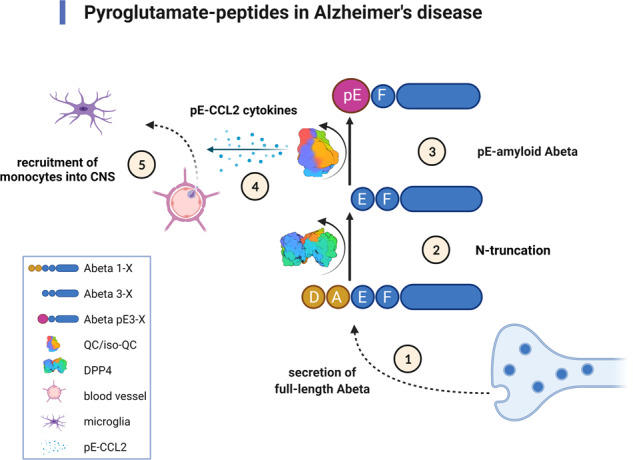
Generation of pyroglutamated (pE) peptides involved in Alzheimer’s disease. ① The N-terminus of full-length Aβ is generated by BACE1 or meprin-β and secreted by neurons. ② Next, N-terminal amino acids are cleaved off by aminopeptidase A (APA), meprin-β or dipeptidyl peptidase 4 (DPP4). ③ Glutamate at position three of the N-terminus of Aβ is subsequently post-translationally modified into N-terminal pyroglutamate (pE) by dehydration catalyzed by glutaminyl cyclase (QC) activity. ④ The isoenzyme of QC, isoQC, predominantly converts the N-terminus of chemokine ligand 2 (CCL2) into pE-CCL2 ⑤ triggering monocyte recruitment into the central nervous system (CNS). The surface structure of DPP4 [84] and of QC [85] was taken from the Protein Data Bank (PDB). Created with BioRender.com.

Figure 1 shows a schematic presentation of the different steps for generation of N-terminal pyroglutamate peptides involved in AD etiology as well as the cellular pathways involved. In the last years, considerable progress was achieved in elucidating the different molecular steps for generation of pyroglutamate-modified Aβ. β-site APP cleaving enzyme 1 (BACE1) is the first and rate-limiting step in the production of full-length Aβ [18] from its precursor the APP [19]. BACE1 cleaves between Met (position −1 of Aβ peptide) and Asp-1 (position +1 of Aβ peptide). This cleavage can also be carried out by meprin-β (reviewed in [20]). Another cleavage site of meprin-β is between Asp-1 and Ala-2 (liberating Aβ_2-*X*_). Besides meprin-β, aminopeptidase A (APA) can trigger the initial first amino acid (Asp-1) cleavage of Aβ [21]. Valverde et al. [22] have convincingly demonstrated that pharmacological reduction of APA activity or lowering of APA expression by shRNA leads to reduced levels of plaque-associated Aβ_pE3-*X*_ and Aβ_1-*X*_ peptides, together with normalized memory deficits in 3xTg-AD mice. Of note, Aβ_pE3-*X*_ in plaques was correlated with APA activity and early Braak stages in brains of patients with sporadic AD. Therefore, APA represents a key enzyme as a potential drug target involved in N-terminal truncation of Aβ peptides independent from dipeptidyl peptidase 4 (DPP4) activity [22].

The enzymatic cleavage between Ala-2 and Glu-3 (liberating Aβ_3-*X*_) is carried out by meprin-β or DPP4 [23, 24]. DPP4 is known to participate for example in cleaving neuropeptide Y [25]. Antonyan et al. [23] have demonstrated that purified DPP4 acts together with glutaminyl cyclase (QC) activity to generate Aβ_pE3-40/42_ in vitro by MALDI-TOF mass spectrometry. Valverde et al. [26] added convincing evidence that human recombinant DPP4 is the rate-limiting enzyme liberating Aβ_3-*X*_ from its precursor Aβ_1-*X*_: (i) Pharmacological treatment with a DPP4-specific inhibitor elevated levels of full-length Aβ on the expense of Aβ_pE3-40_. (ii) Lentiviral expression of mutant APP cDNA in wild-type mouse brain and pharmacological blocking of DPP4 activity in brain in situ rescued dendritic spine morphology. (iii) This was further supported by studies using the 3xTg-AD mouse model. Amyloid-plaque load was reduced and memory deficits were normalized in this model by pharmacological and shRNA-driven inhibition of DPP4 activity. (iv) In addition, DPP4 activity seemed to be transiently elevated during AD pathogenesis. Hence, DPP4 may be a potential drug target against AD [27] and cognitive benefits in patients suffering from diabetes [28]. DPP4 is long known for treating diabetes as it metabolizes glucagon-like peptide-1 [29].

There is considerable evidence that QC is the rate-limiting enzyme in the final step the conversion of N-truncated Aβ_3-*X*_ into pyroglutamate Aβ_pE3-*X*_ [23, 30–35]. Besides, the normal function of QC to stabilize hormones, peptides, and proteins it may also contribute to neurodegenerative disorders, systemic inflammatory diseases and certain types of oncological conditions (reviewed recently [36]). In the 5XFAD mouse model for AD, overexpression of human QC and knock-out of murine QC clearly demonstrated that memory function correlated well with the formation of Aβ_pE3-*X*_ [32]. Although complete loss of QC activity in homozygous QC knock-out mice did not eliminate Aβ_pE3-*X*_ levels completely suggesting that there is a resting QC-like activity by other enzyme(s). Such an activity has been added by Cynis et al. [37] demonstrating that the isoenzyme of QC (isoQC) predominantly modulated chemokine ligand 2 (CCl2; synonyms: monocyte chemotactic protein 1 and small inducible cytokine A2) thereby fostering pE-CCL2 formation and monocyte infiltration. Moreover, pharmacological inhibition of QC/isoQC-activity reduced the pathology in a mouse model for atherosclerosis. The function of QC/isoQC activity in the etiology of AD addresses directly the generation of toxic Ab_pE3-*X*_ peptides, but equally important may modulate microglia function via recruitment of monocytes from the periphery. Of note, the dual function of microglia cells in AD pathology should be taken into account. They participate in both beneficial amyloid-β clearance, but also in destructive inflammation in AD brain (reviewed in detail [38]).

## Properties of pyroglutamate Aβ

Saido et al. [39] hypothesized that the lactam ring of Aβ_pE3-*x*_ peptides and loss of two negative and one positive charges leads to higher hydrophobicity, higher peptide stability, increased aggregation propensity and may escape enzymatic degradation. Another twist in the complex story was published by Nussbaum et al. [40], who pointed out that Aβ_pE3-42_ peptides can form soluble oligomers that potentially seed full-length Aβ_1-42_ and are toxic in a tau-dependent manner. Cross-seeding activities of Aβ_pE3-*X*_ peptides with wild-type Aβ_1-*X*_ were also reported by Hu et al. [41, 42]. In addition, Aβ_pE3-42_ and Aβ_4-42_ when expressed together elicited neurological deficits, which were significantly stronger as compared to expression of Aβ_pE3-42_ or Aβ_4-42_ alone [43]. Neuronal expression of Aβ_pE3-42_ triggered amyloid-plaque load and memory deficits when crossed to amyloid-plaque AD mouse models [44, 45].

The secondary structure of Aβ_pE3-42_, Aβ_4-42_ and Aβ_1-42_ peptides was analyzed by far-UV circular dichroism (CD) spectroscopy demonstrating that the CD spectra of monomers were characteristic of a disordered conformation [46]. The aggregation propensities of the Aβ variants analyzed by liquid-state nuclear magnetic resonance (NMR) spectroscopy suggested that the temporal loss of signal intensities was due to conversion of NMR-visible monomers and small aggregates into larger aggregates. The highest loss in signal intensity was found for N-truncated Aβ. Hence, it was reported that Aβ_pE3-42_ and Aβ_4-42_ rapidly formed aggregates with a high aggregation propensity in terms of monomer consumption and oligomer formation [46]. Acute treatment of primary cortical neurons indicated that Aβ_pE3-42_ and Aβ_4-42_ are equally toxic as Aβ_1-42_. This was further corroborated by induction of working memory deficits after intraventricular injection of Aβ_pE3-42_, Aβ_4-42_ or Aβ_1-42_ in wild-type mice [46, 47].

In aqueous solution and in 10% sodium dodecyl sulfate micelles Aβ_pE3-40_ peptides showed increased β-sheet formation using CD spectroscopy and aggregation behavior by sedimentation analysis when compared with Aβ_1-40_ [48]. The relative toxicity and abundance of N-truncated and full-length Aβ variants both in vitro and in vivo in AD mouse models was reviewed and discussed previously [21, 49–51].

## Drug target pyroglutamate Aβ

**Fig. 2:**
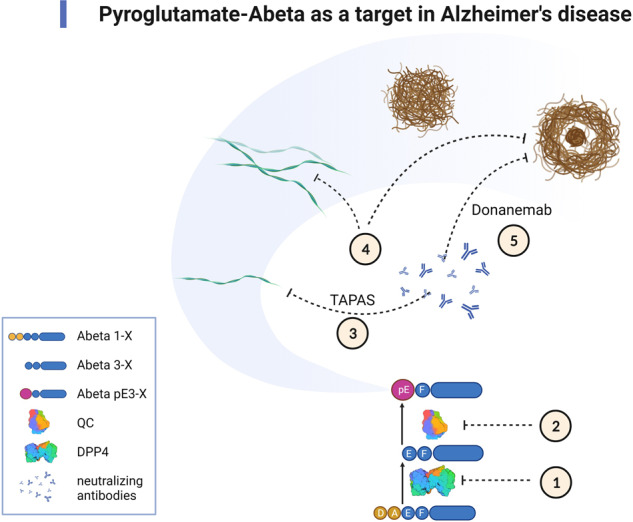
Pyroglutamate Aβ as a potential drug target against Alzheimer’s disease. ① Inhibition of DPP4 prevents N-terminal truncation of the first two amino acid residues of the full-length Aβ monomers (Aβ_1-*x*_). ② Inhibition of QC activity prevents the conversion of Aβ_3-*x*_ into Aβ_pE3-*x*_ monomers. ③–⑤ Neutralizing antibodies react with different conformational and structural variants of Aβ_pE3-*X*_. ③ Once Aβ_pE3-*x*_ monomers are generated, they adopt a pseudo β-hairpin structure at the N-terminus, which is specifically recognized by the TAPAS family of antibodies. The pseudo β-hairpin epitope is neutralized by the TAPAS vaccine and by TAPAS monoclonal antibodies. ④ Pan-Aβ_pE-*X*_ antibodies react with a range of conformations: soluble oligomers, protofibrils and fibrillar forms found in different plaque types. ⑤ Donanemab, a plaque-specific monoclonal antibody, reacts with Aβ_pE3-*X*_ aggregates found in the amyloid-plaque cores. The surface structure of DPP4 [84] and of QC [85] was taken from the Protein Data Bank (PDB). Created with BioRender.com.

The different steps for therapeutic intervention against the pyroglutamate amyloid-β cascade in the etiology of AD are shown in Fig. 2. It is generated by a two-step process starting by a first cut of the APP between Met at postion-1 and Asp at position +1 by β-site APP cleaving enzyme 1 (BACE1), which generates the N-terminus of full-length Aβ_1-*X*_ [33]. In addition, meprin-β can cut between Met-1 and Asp+2 independent from BACE1 [20]. DPP4 is responsible for the cleavage of the first two N-terminal amino acids of full-length Aβ_1-*X*_ generating Aβ_3-*X*_ [23, 26]. There is one clinical study published that investigated the influence of DPP4 inhibition in patients with dementia [52]. In this retrospective clinical study the effect of vildagliptin, a DPP4 inhibitor, was investigated on cognitive dysfunction in 60 elderly patients with diabetes with additional diagnosis of mild cognitive impairment (MCI). Fifty percent of the patients were treated with metformin as standard medication control; the other 50% received metformin plus vildagliptin. A significant beneficial effect on stabilizing cognitive scores was observed in the DPP4 inhibitor group [52]. This pilot study did not include however, the assessment of AD biomarkers commonly employed in clinical trials with MCI patients. In addition, it is not clear whether pharmacological inhibition of DPP4 activity is clinically meaningful in nondiabetic MCI and AD patients.

Alternatively, and independent from DPP4 activity, a sequential cleavage of full-length Aβ between Asp at position +1 and Ala at position +2 may also occur by aminopeptidase A activity thereby influencing formation of Aβ_pE3-*X*_ [21, 22].

The next step in the cascade is the conversion of N-terminal Glu-3 of Aβ into Aβ_pE3-*X*_ by QC. In preclinical experiments, pharmacological QC inhibition had significant beneficial effects on Aβ_pE3_ levels, plaque load, gliosis and memory function [33]. PQ912, a competitive inhibitor of QC, was analyzed in clinical trials in order to assess the safety, pharmacokinetics, and pharmacodynamics in healthy individuals [53], MCI and AD patients [54, 55] and was reported to be safe. QC-dependent blood biomarker levels were significantly altered by treatment with PQ912.

The conformation of the structure of amyloid-β fibrils is well established eliciting a cross-β structure identified by x-ray diffraction analysis [56]. The individual β-strands of amyloid fibrils are oriented perpendicular to the fibril axis [56]. NMR spectroscopy revealed that the N-terminus of full-length Aβ_1-42_ is unstructured [57, 58]. Ahmed et al. [59] reported that full-length Aβ oligomers are more toxic to mouse cortical than protofibrils and fibrils. Of note, these oligomers are composed of loosely aggregated strands and do not have the β-sheet structure characteristic of fibrillar amyloid-β.

Therefore, it is of upmost interest that the N-terminus of Aβ_pE3-*X*_ monomers adopt a unique pseudo β-hairpin structure, which is recognized by the TAPAS family antibodies (murine and humanized variants) [60]. A stabilized cyclic form of Aβ_1-14_ (N-truncated amyloid peptide antibodies, the “TAPAS” vaccine) was engineered and shown that the vaccine adopted the same conformation as the native sequence when bound to TAPAS antibodies. Active immunization of two AD mouse models with the TAPAS vaccine led to a significant reduction in amyloid-plaque load, a rescue of brain glucose metabolism, rescue of neuron loss, and rescue of memory deficits. Both, active immunization with the TAPAS vaccine and passive immunization with the humanized TAPAS version of the murine precursor antibody [61, 62] had similar treatment effects. Of note the TAPAS binding motif is not found in amyloid plaques [60, 63].

The monoclonal antibody 9D5 reacted with low molecular weight oligomers (4–10 mers) of Aβ_pE3-*X*_ and not with amyloid plaques in AD brain tissue [64]. Passive immunization of 5XFAD mice reduced amyloid plaques and rescued behavioral deficits.

Other antibodies preferentially react with amyloid plaques from AD patients, like the monoclonal antibody 3B8, which is specific for both conformational epitopes present on N-terminal truncated Aβ_pE3-42_ and its unmodified counterpart Aβ_3-42_ [65]. Another report supports the concept that pan-Aβ_pE-*X*_ antibodies preferentially bind to amyloid plaques in brain tissue of sporadic and familial AD cases [14]. The Aβ_pE3-*X*_ specific antibody 07/01 detected diffuse and senile plaques and cerebral amyloid angiopathy in the brain of human specimen and in nonhuman primates, as well as in a subset of focal and vascular deposits in transgenic mouse models for AD [66].

BAMB31 is another pan-Aβ_pE_ monoclonal antibody designed to target plaques. Passive immunization elicited therapeutic effects in an acute application design using the APPPS1 mice [67]. Chronic cotreatment with a BACE1 inhibitor cleared preexisting plaques in APP_Lon_ and PDAPP mouse models of AD. The authors report that the plaque lowering effect was not accompanied by microhemorrhages.

Passive immunization with the monoclonal antibody 07/01 reduced plaque load and normalized behavioral deficits in the APPswe/PS1DeltaE9 model for AD [68, 69]. The 07/01 antibody was compared with other Aβ_pE3-*X*_ antibodies using structural and functional assays. The authors reported that only 07/01 prevented in vitro toxicity of Aβ_pE3-42_ oligomers [70]. Furthermore, surface plasmon resonance revealed that all Aβ_pE3-*X*_ antibodies preferentially reacted with amyloid fibrils. Enhanced phagocytosis of amyloid plaques could be achieved without inducing neuroinflammation using in vivo PET imaging in the APPSLxhQC model for AD [71]. The humanized antibody, PBD-C06, retained the binding properties, including mixed aggregates of full-length Aβ and Aβ_pE3-*X*_ peptides [72].

Considerable attention in the AD field was raised by the publication of the results of the TRAILBLAZER-ALZ trial. The phase 2 trial evaluated safety, tolerability and efficacy of passive immunization with donanemab a plaque-specific humanized antibody against Aβ_pE3-*X*_ [73]. The study design was unique, as the screening of early symptomatic AD patients was based on a tau threshold screening by brain imaging with flortaucipir PET scanning to assess tangle deposition in vivo. Only patients with intermediate levels of tangle formation were subsequently enrolled in the study. The primary outcome measures were tests to assess cognitive function. The secondary outcome measures assessed in addition amyloid-plaque load and tangle deposition by appropriate PET scans. Donanemab significantly slowed the disease process, slowed cognitive and functional decline on all secondary clinical endpoints, and reduced plaque load, and tau accumulation in a subgroup of patients. Of note, patients with the lowest tau accumulation demonstrated the highest benefit, while patients with the highest tau accumulation did not benefit at all. The safety profile was similar to findings of the phase 1 trial [74]. Although the major endpoints of the phase II donanemab trial were reached and are generally promising, the outcome on the neuropathological level is not entirely clear. For example, neurodegeneration and tau pathology progression were slowed down, but not reversed. Therefore, it is not entirely proven whether Aβ_pE3-*X*_ directly triggers tau pathology. One needs to bear in mind, that many neuropathological studies have demonstrated that tau pathology precedes plaque pathology (for example [75, 76]). Of note, the sensitivity of biomarkers used in clinical studies is still lower in comparison with that of post-mortem neuropathological evaluations [77–82].

The meaning of the data of the TRAILBLAZER-ALZ is a matter of ongoing scientific debates as aggregated fibrillar amyloid is generally thought to be pathologically inert as discussed above [59]. Ackley et al. [83] added further evidence to the discussion. The authors reported on a meta-analysis of randomized controlled clinical trials of drugs for the prevention or treatment of AD targeting amyloid mechanism. They concluded that amyloid-plaque reduction strategies did not substantially improve cognition.

In conclusion, there is ample evidence that pyroglutamate Aβ is involved in the etiology of AD. The pyroglutamate amyloid-β cascade provides several attractive therapeutic intervention points. DPP4 or QC inhibitors can modulate the stepwise formation of pyroglutamate Aβ enzymatically. Pyroglutamate Aβ induced toxicity can be neutralized by antibodies specific for the TAPAS epitope formed by the N-terminus of monomers or by antibodies against amyloid aggregates formed by oligomers, protofibrils and other high-molecular weight structures.
